# Comparison of Dried Plum Puree, Rosemary Extract, and BHA/BHT as Antioxidants in Irradiated Ground Beef Patties

**DOI:** 10.1155/2013/360732

**Published:** 2013-05-23

**Authors:** Iulia Movileanu, Máryuri T. Núñez de González, Brian Hafley, Rhonda K. Miller, Jimmy T. Keeton

**Affiliations:** ^1^Department of Neurology, Upstate Medical University, 812 Jacobsen Hall, Syracuse, NY 13210, USA; ^2^Department of Food Technology, Universidad de Oriente, Núcleo Nueva Esparta, Escuela de Ciencias Aplicadas del Mar, Isla de Margarita 6301, Venezuela; ^3^Tyson Foods, 1825 Ford Avenue, Springdale, AR 72764, USA; ^4^Department of Animal Science, Texas A&M University, 338 Kleberg Center, College Station, TX 77843-2471, USA; ^5^Department of Nutrition and Food Science, Texas A&M University, 122 Kleberg Center, College Station, TX 77843-2253, USA

## Abstract

Fresh ground beef patties with (1) no antioxidant (control), (2) 0.02% butylated hydroxyanisole/butylated hydroxytoluene (BHA/BHT), (3) 3% dried plum puree, or (4) 0.25% rosemary extract were aerobically packaged, irradiated at target doses of 0, 1.5, or 2.0 kGy (1.7 and 2.3 kGy actual doses), and stored at 4°C. The samples were evaluated for lipid oxidation on 0, 3, 7, 14, 21, and 28 days of storage after irradiation. When compared to the control, all antioxidant treatments were effective in retarding (*P* < 0.05) irradiation-induced lipid oxidation during storage as determined by 2-thiobarbituric acid reactive substances (TBARs) values. Rosemary extracts had the same antioxidant effect (*P* > 0.05) as BHA/BHT in irradiated and nonirradiated beef patties, followed by the dried plum puree treatment. Irradiation increased TBARs values, but no differences were noted in oxidation between irradiation dose levels.

## 1. Introduction

Irradiation of refrigerated and frozen red meat and poultry has been approved by the Food and Drug Administration (FDA) [1] and the United States Department of Agriculture-Food Safety and Inspection Service (USDA-FSIS) at levels up to 4.5–7.0 kGy and 3.0 kGy, respectively, to effectively reduce or eliminate pathogenic bacteria [2]. Recently, the FDA approved a maximum absorbed dose of ionizing radiation of 4.5 kGy to treat unrefrigerated (as well as refrigerated) uncooked meat, meat by-products, and certain meat food products to reduce levels of foodborne pathogens and extend shelf-life. Also, the regulation indicates that irradiation is a safe treatment for fresh (refrigerated and unrefrigerated) poultry food products and frozen poultry products at absorbed doses that do not exceed 4.5 kGy and 7.0 kGy, respectively [3]. This technology can eliminate foodborne pathogenic microorganisms in meats and help provide a safer food supply for human consumption [4]. Populations of the most common enteric pathogens such as *Escherichia coli *O157:H7*, Staphylococcus aureus, Salmonella, Listeria monocytogenes, *and *Aeromonas hydrophila *can be significantly decreased or eliminated by low dose (<3.0 kGy) treatments with ionizing radiation [5].

Use of irradiation on raw meat, however, results in the formation of radiolytic products [6] that can cause oxidation of myoglobin and lipids leading to discoloration, lipid oxidation, and formation of off-odor and off-flavor compounds [7, 8]. Free radicals and hydroperoxides generated by irradiation can also destroy endogenous antioxidants, reduce storage stability, and increase off-flavors from lipid and protein degradation [9, 10]. Although anaerobic packaging is an effective means of reducing oxidation, incorporation of antioxidants, synthetic or natural, is one of the major strategies for preventing lipid oxidation [11] and improving color stability of meat products. To be effective, antioxidants must compete with reactive meat components for free radicals generated by lipid oxidation and irradiation or inhibit the formation of free radicals induced by prooxidative metals [12, 13].

Rosemary (*Rosmarinus officinalis*) extracts have been found to be effective in inhibiting lipid oxidation in meat systems primarily through the presence of phenolic compounds [14, 15] which break free radical chain reactions by hydrogen atom donation [16]. Rosemary and rosemary extracts at concentrations ranging from 0.02% to 1% have been perhaps the most investigated natural antioxidants in meat products [17–21]. Various authors have reported the effectiveness of rosemary in ground beef [22], beef patties [23–26], pork patties [20, 27], pork sausage [28], and beef steaks [29]. 

Previous research has shown that dried plums have one of the highest antioxidant activities of both fruits and vegetables [30]. The principal phytochemicals in dried plums are phenolic compounds. These appear to be the primary contributors to antioxidant properties, are responsible for inhibition of low-density lipoprotein oxidation [31–33], and may play a role in suppressing cellular inflammation, a precursor to cancer development. The native antioxidant properties of dried plums may offer alternatives to the meat industry based on their potential to reduce microbial growth, retain moisture, and prevent off-flavor formation in meat [34].

The addition of 3% plum extract has been shown to improve mouthfeel and the antioxidant properties in irradiated turkey breast rolls [35]. Núñez de González et al. [36] demonstrated that dried plum puree at levels of 3% to 6% in precooked pork sausage was as effective as a butylated hydroxyanisole/butylated hydroxytoluene (BHA/BHT) combination in suppressing lipid oxidation. Moreover, Núñez de González et al. [37] reported that 2.5% fresh juice concentrate or dried plum juice concentrate could be incorporated into beef roasts to reduce lipid oxidation and potentially warmed-over flavor (WOF). Thus, the use of naturally occurring antioxidants (or extractives) with high antioxidant capacity may prove useful in retarding or reducing off-odors and off-flavors in irradiated meat products and particularly ground beef patties which are more prone to lipid oxidation.

Synthetic antioxidants such as butylated hydroxyanisole (BHA) and butylated hydroxytoluene (BHT) have been widely used in meat products [25, 28, 36, 38]. USDA regulations permit up to 0.01% (based on fat content) each of BHA and BHT in fresh sausage and up to 0.003% (based on total weight) each in dry sausage [39]. Addition of 0.02% BHA/BHT (0.01% BHA + 0.01% BHT) in ground beef has been shown to be more effective in retarding lipid oxidation, as determined by lower levels of 2-thiobarbituric acid reactive substances (TBARs) [19], when compared to natural plant extracts. TBARs values for the treated samples did not change significantly during storage. 

The objective of this study was to compare the antioxidant effectiveness of dried plum puree (3%), BHA/BHT (0.02%), and rosemary extract (0.25%) in irradiated (0, 1.5, or 2.0 kGy) ground beef patties stored refrigerated over a 28-day period to assess their capacity in retarding lipid oxidation as measured by TBARs. 

## 2. Materials and Methods 

### 2.1. Beef Patty Preparation

Approximately 130 kg of fresh, coarse ground (1.27-cm plate) beef lean and fat trimmings were purchased from Ruffino Meats, Inc. (Bryan, TX), 3 to 4 d postmortem. Four 27.3 Kg batches were formulated to contain 20% fat and assigned to treatments of no antioxidant (control), BHA/BHT (0.02% combination based on the fat content, crystalline TENOX, Eastman Chemical Products, Kingsport, TN), rosemary extract (0.25% based on batch weight, liquid oil based HERBALOX, Type HT 25, Kalsec, Inc., Kalamazoo, MI), and dried plum puree (3% based on batch weight, Sunsweet Growers Inc., Yuba City, CA). No additional ingredients were included in the control treatment so that the true irradiation effect on 100% ground beef could be measured. The level of dried plum puree incorporated into ground beef patties was determined from a previous study in which 3% was found to be effective in retarding lipid oxidation in precooked pork sausage [36]. Moreover, Lee and Ahn [35] indicated that the addition of 3% plum extract was recommended to improve mouthfeel and antioxidant effect in irradiated turkey breast rolls.

 Beef patties were manufactured in a state-inspected (Texas Department of Health) commercial-scale pilot plant located in the Rosenthal Meat Science and Technology Center at Texas A&M University. Each coarse-ground treatment was blended in a paddle mixer (Model 150 767, Butcher Boy, Lasar, MFG Inc., Los Angeles, CA) for 3 min and reground through a 0.635-cm plate (Biro Model 1056 22772, The Biro Mfg Co., Marblehead, OH). Dried plum puree, rosemary extract, and BHA/BHT were incorporated directly into the mixer in their original commercial form. Patties (113 g, 10.5 cm diameter, 1 cm thick) were made (Hollymatic JET FLOW Super Food Portioning Machine, 1033, Hollymatic Corp., Park Forest, IL) and stored at −40°C for 2 h. Approximately 220 patties per treatment were produced in this study. The crust frozen patties were then placed in Cryovac bags (Cryovac, Sealed Air Corporation, Duncan, SC) and aerobically packaged (a vacuum setting 3.5 on a 10 point scale where 10 = maximum vacuum) to a constant level of air using an Ultravac packaging machine (Model 2100 D, KOCH Inc, Kansas City, MO). An aerobic package environment was chosen to exacerbate lipid oxidation and challenge the antioxidant treatments when exposed to a constant concentration of air and two electron-beam irradiation levels. Pulling a full vacuum (setting of 10) would have reduced irradiation mediated oxidation of each treatment and would have reduced the oxidative environment to stress each antioxidant's capacity for suppressing oxidation. After packaging, the patties were rapidly frozen (−40°C) prior to shipping to an electron beam irradiation facility.

### 2.2. Irradiation of Samples and Storage

Frozen patties were placed in insulated containers with ice packs and shipped overnight to the Surebeam Corporation irradiation facility (Glendale Heights, IL). Samples were tempered and irradiated at −4°C at targeted doses of 0 (control), 1.5, and 2 kGy while passing through a linear accelerator. The targeted doses were determined based on previous studies [40, 41]. For each batch, a dosimetry profile was established for uniform packages of 12 patties stacked three high with an actual mean dose level of 1.7 and 2.3 kGy, respectively, for the irradiation treatments. To confirm the target dose, 2 alanine dosimeters per cart were attached to the top and bottom surface of a sample. All samples were hard frozen (−20°C) after irradiation and returned via overnight carrier to the Department of Animal Science at Texas A&M University. The patties remained in their original package as described above and were then stored and refrigerated at 4°C and evaluated on storage days 0, 3, 7, 14, 21, and 28. Day 0 was designated the next day after the return of the irradiated samples.

### 2.3. 2-Thiobarbituric Acid Reactive Substances (TBARs)

 Lipid oxidation of the ground beef patties from each treatment, irradiation level, and storage day was determined using the distillation 2-thiobarbituric acid (TBA) procedure of Tarladgis et al. [42], with the addition of propyl gallate and ethylenediaminetetraacetic acid (EDTA) at the sample blending step to prevent further lipid oxidation [43]. TBARs determinations were performed in duplicate, and results were expressed as mg of malonaldehyde per kg of meat sample. The percent reduction in TBARs by different treatments in comparison to control was also determined.

### 2.4. Experimental Design and Statistical Analysis

The experiment was designed as a factorial arrangement with a split-plot design. Batch treatments included (1) control (no antioxidant); (2) BHA/BHT; (3) dried plum puree, and (4) rosemary extract. The patties were aerobically packaged, irradiated at actual levels of 0, 1.7, and 2.3 kGy, and stored at 4°C for 0, 3, 7, 14, 21, and 28 days. Seventy-two patties were randomly assigned to the batch treatment, dose level and storage condition (Table 1). One trial replication was processed to establish shipping requirements and analytical logistics followed by three complete experimental replications that were conducted individually approximately 6-7 weeks apart. 

Analysis of variance was conducted using the General Linear Model (GLM) procedure of statistical analysis system [44]. Data was analyzed as a factorial arrangement with a split-plot design. The whole-plot included the main effects of batch and treatment. The error term for the whole plot was the batch by treatment interaction. The split-plot included the main effects of radiation dose and storage days and their interactions. The residual error was used to test split-plot effects at a significance value of *P* < 0.05. Least square means were separated using the PDIFF statement of GLM [44]. Least square means were generated and reported for the main effects and for significant interactions. 

## 3. Results and Discussion

TBARs values of beef patties containing different antioxidants and irradiated at two dose levels over 28 days at refrigerated conditions are presented in Tables 2 and 3. No differences in TBARs values (*P* < 0.05) were noted between doses 1.7 and 2.3 kGy, regardless of antioxidant treatment (Table 2). The inclusion of BHA/BHT or rosemary extract retarded the oxidative process regardless of irradiation dose level, and TBARs values were lower than control patties not receiving irradiation. Lee et al. [38] found that thiobarbituric acid values were not very different during storage regardless of the irradiation dose or the type of antioxidant (rosemary extracts and BHA). In the present study, irradiated samples without antioxidants (control) had significantly higher TBARs values (*P* < 0.05) than the 1.7 and 2.3 kGy irradiation levels with antioxidants (Table 2). Previous studies have indicated that irradiation accelerated lipid oxidation of raw meat under aerobic conditions [13, 40, 45]. Nawar [46] reported that irradiation in the presence of oxygen accelerated the autoxidation of fats by one of three possible reactions: (1) formation of free radicals which combine with oxygen to form hydroperoxides; (2) breakdown of hydroperoxides; and/or (3) destruction of antioxidants. 

TBARs values showed that dried plum puree lessened the effect of irradiation-induced oxidation but also showed that it was less effective than BHA/BHT or rosemary treatments (Table 2). Overall, TBARs values were higher for dried plum puree than BHA/BHT or rosemary extract, even though dried plum puree partially reduced the oxidative effects of irradiation. It is postulated that the antioxidant component(s) in 3% dried plum puree might not be as concentrated as that found in the rosemary extract or BHA/BHT. Thus, equivalent levels of the antioxidant components in dried plum puree need to be identified and characterized prior to additional comparisons. The protective antioxidant effect of BHA/BHT and rosemary extract was not altered by irradiation dose. However, it cannot be ruled out that oxidation might occur in beef patties treated with higher levels of irradiation. In conclusion, rosemary extract and BHA/BHT treatments maintained lower and equivalent TBARs values regardless of dose level (1.7 or 2.3 kGy), while dried plum puree patties had slightly higher TBARs values. These values, however, were still significantly lower (*P* < 0.05) than the irradiated control.

 All antioxidant treatments reduced TBARs values of all irradiated samples during the storage period when compared to the control (Table 3). Lipid oxidation, as measured by TBARs, of the control treatment increased significantly (*P* < 0.05) through day 7, remained static to day 21, and increased slightly from day 21 to day 28. Among the antioxidant treatments, samples containing BHA/BHT and rosemary extract showed the greatest antioxidant effect. There was an increase in TBARs values over time for the dried plum puree, but not to the same degree as the control. This increase was likely due to the inability of the dried plum puree's antioxidant component to completely suppress the oxidative effect of irradiation. Although, BHA/BHT and rosemary extract TBARs values increased slightly numerically, they were not statistically (*P* > 0.05) significant within a treatment over the storage period. When compared to the control, BHA/BHT and rosemary reduced TBARs by 69 and 67%, respectively, at day 0 (Table 4) and continued to have consistently high antioxidant activity throughout the 28-day storage period. Nam and Ahn [13] compared the TBARs values of irradiated pork homogenates containing sesamol (200 *μ*M) and Trolox (200 *μ*M) and found them to reduce TBARs values by as much as 72% when compared to other antioxidants after 5 days of storage. Nam et al. [12] indicated that irradiated pork loins treated with rosemary-tocopherol/double packaging had lower TBARs values than a vacuum-packaged control after 10 days of refrigerated storage. Rosemary oleoresin (200 ppm based on fat content) in pork patties, irradiated at 4.5 kGy and stored at 4°C for 7 days, was shown to be effective only for the first 3 days of the storage period [9], while BHT (0.01%) was effective across all storage days.

 In this study, BHA/BHT and rosemary TBARs values did not differ (*P* > 0.05) from one another during storage of irradiated beef patties. Similarly, rosemary extract was noted to be equally effective as BHA/BHT in maintaining low TBARs values of nonirradiated refrigerated, fresh pork sausage and cooked-frozen sausage [28]. McBride et al. [25] indicated that rosemary extract was more effective in controlling lipid oxidation in aerobically stored beef samples as compared with a control or a BHA/BHT treatment. Additional antioxidant effects of rosemary in meats and meat products have been reported extensively in the scientific literature [20, 22–24, 26, 29].

 Dried plum puree was not as effective as rosemary extract or BHA/BHT (Tables 3 and 4) in inhibiting oxidation over the storage period but did reduce (*P* < 0.05) TBARs values by approximately 50% when compared to the control. At day 0 (Table 4), dried plum puree decreased TBARs values by 47.4% but was less effective as an antioxidant from day 14 to 21. Lee and Ahn [35] likewise reported that the addition of 2% or 3% of plum extract puree was effective in controlling lipid oxidation in irradiated turkey breast rolls.

 Dried plum puree contains numerous components such as dextrose, sorbitol, and other compounds that could dilute and lessen its antioxidant effectiveness. Based on this observation, further work is needed to extract, identify, and standardize the principle antioxidant components of dried plum puree and then test their efficacy. Thus, a dried plum puree extract could potentially have the same or possibly greater antioxidant capacity as that of rosemary extract or a BHA/BHT combination in irradiated ground beef patties.

## 4. Conclusions

All antioxidant treatments decreased TBARs values in ground beef patties irradiated at 1.7 and 2.3 kGy. Rosemary (0.25%) and BHA/BHT (0.02%) were the most effective antioxidants, while dried plum puree (3%) also reduced lipid oxidation but to a lesser degree. Irradiation doses of 1.7 and 2.3 kGy increased TBARs values, but no differences in oxidation were noted between irradiation dose levels.

## Figures and Tables

**Table 1:** Experimental design^b^ of patty allocation by treatment and storage time.

Treatment		Storage time (day)						
	Actual irradiation dose (kGy)	0	3	7	14	21	28	Total
	0	12	12	12	12	12	12	72
Control^a^	1.7	12	12	12	12	12	12	72
	2.3	12	12	12	12	12	12	72

	0	12	12	12	12	12	12	72
BHA/BHT^a^	1.7	12	12	12	12	12	12	72
	2.3	12	12	12	12	12	12	72

	0	12	12	12	12	12	12	72
Dried plum puree^a^	1.7	12	12	12	12	12	12	72
	2.3	12	12	12	12	12	12	72

	0	12	12	12	12	12	12	72
Rosemary extract^a^	1.7	12	12	12	12	12	12	72
	2.3	12	12	12	12	12	12	72

Total								864

^a^Control = no antioxidant; BHA/BHT = butylated hydroxyanisole/butylated hydroxytoluene (0.02% of fat content); dried plum puree = 3.0% based on batch weight; rosemary extract = 0.25% based on batch weight.

^b^A total of three replications were performed in this experiment.

**Table 2:** Least square means for TBARs values of ground beef patties with/without antioxidants and irradiated at two dose levels.

Treatment^d^	Dose^e^ (kGy)	TBARs (mg malonaldehyde/kg sample)
	0	1.56^b^
Control	1.7	2.70^c^
	2.3	2.68^c^
	0	0.42^a^
BHA/BHT	1.7	0.37^a^
	2.3	0.52^a^
	0	0.43^a^
Dried plum puree	1.7	1.60^b^
	2.3	1.61^b^
	0	0.57^a^
Rosemary extract	1.7	0.47^a^
	2.3	0.48^a^
RMS^f^		0.55

^a–c^Least square means in the same column with superscripts that differ are significantly different (*P* < 0.05).

^d^Control = no antioxidant; BHA/BHT = butylated hydroxyanisole/butylated hydroxytoluene (0.02% of fat content); dried plum puree = 3.0% based on batch weight; rosemary extract = 0.25% based on batch weight.

^e^Actual dosimetry levels were 1.7 and 2.3 kGy.

^f^RMS = root mean square error.

**Table 3:** Least square means for TBARs values of irradiated ground beef patties treated with/without antioxidants and stored at 4°C for 28 days.

Treatment^h^	Storage day	TBARs (mg malonaldehyde/kg sample)
Control	0	0.97^bcd^
	3	1.97^e^
	7	2.52^f^
	14	2.79^fg^
	21	2.58^fg^
	28	3.04^g^
BHA/BHT	0	0.30^a^
	3	0.32^a^
	7	0.36^a^
	14	0.44^a^
	21	0.74^abc^
	28	0.46^ab^
Dried plum puree	0	0.51^ab^
	3	0.83^abc^
	7	1.02^cd^
	14	1.56^e^
	21	1.89^e^
	28	1.46^de^
Rosemary extract	0	0.32^a^
	3	0.39^a^
	7	0.44^a^
	14	0.54^abc^
	21	0.53^abc^
	28	0.83^abc^
RMS^i^		0.55

^a–g^Least square means in the same column with superscripts that differ are significantly different (*P* < 0.05).

^h^Control = no antioxidant; BHA/BHT = butylated hydroxyanisole/butylated hydroxytoluene (0.02% of fat content); dried plum puree = 3.0% based on batch weight; rosemary extract = 0.25% based on batch weight.

^i^RMS = root mean square error.

**Table 4:** Percent decrease in TBARs values for irradiated ground beef patties treated with/without antioxidants and stored at 4°C for 28 days.

	Percent decrease in TBARs as compared to control^a^					
Treatment^b^	Storage day					
	0	3	7	14	21	28
BHA/BHT	69.1	83.8	85.7	84.2	71.3	84.9
Rosemary extract	67.0	80.2	82.5	80.6	79.4	72.7
Dried plum puree	47.4	57.9	59.5	44.0	26.7	52.0

^a^Data not statistically analyzed, but percentages were computed from the statistical data in Table 3.

^b^BHA/BHT = butylated hydroxyanisole/butylated hydroxytoluene (0.02% of fat content); dried plum puree = 3.0% based on batch weight; rosemary extract = 0.25% based on batch weight.
